# 4-Phenyl-1-(prop-2-yn-1-yl)-1*H*-1,5-benzodiazepin-2(3*H*)-one

**DOI:** 10.1107/S1600536811027371

**Published:** 2011-07-23

**Authors:** Mohamed Loughzail, José A. Fernandes, Abdesselam Baouid, Mohamed Essaber, José A. S. Cavaleiro, Filipe A. Almeida Paz

**Affiliations:** a Laboratoire de Chimie Moléculaire, Département de Chimie, Faculté des Sciences - Semlalia, BP 2390, Université Cadi Ayyad, 40001, Marrakech, Morocco; b Department of Chemistry, University of Aveiro, CICECO, 3810-193, Aveiro, Portugal; c Department of Chemistry, University of Aveiro, QOPNA, 3810-193, Aveiro, Portugal

## Abstract

4-Phenyl-1*H*-1,5-benzodiazepin-2(3*H*)-one reacts in the pres­ence of a concentrated aqueous solution of sodium hydroxide and a quaternary ammonium salt (as catalyst) in benzene (phase transfer catalysis) with propargyl bromide, affording the title benzodiazepine derivative, C_18_H_14_N_2_O. In the mol­ecule, the mean plane of the propargyl substituent is almost perpendicular with that of the amide group [dihedral angle = 87.81 (8)°]. In the crystal, the molecules are linked by C—H⋯O and C—H⋯N inter­actions.

## Related literature

For general background to applications of benzodiazepines, see: Ahmed *et al.* (1983 ▶); Bird (1996 ▶); Di Braccio *et al.* (1990 ▶, 2001 ▶); Goetzke *et al.* (1983 ▶); Kavita *et al.* (1988 ▶); Sieghart & Schuster (1984 ▶); Wolff (1996 ▶). For examples of benzodiazepines used as medicine, see: Wolff (1996 ▶). For the pharmacological effects of benzodiazepines, see: Meldrum & Chapman (1986 ▶). For examples of synthetic pathways of new benzodiazepines, see: Aatif *et al.* (2000 ▶); Baouid *et al.* (2001 ▶); Boudina *et al.* (2007 ▶); Nardi *et al.* (1973 ▶). For previous work from our groups on organic crystals, see: Fernandes *et al.* (2011 ▶); Amarante, Figueiredo *et al.* (2009); ▶ Amarante, Gonçalves & Almeida Paz (2009); ▶ Paz & Klinowski (2003); ▶ Paz *et al.* (2002) ▶.
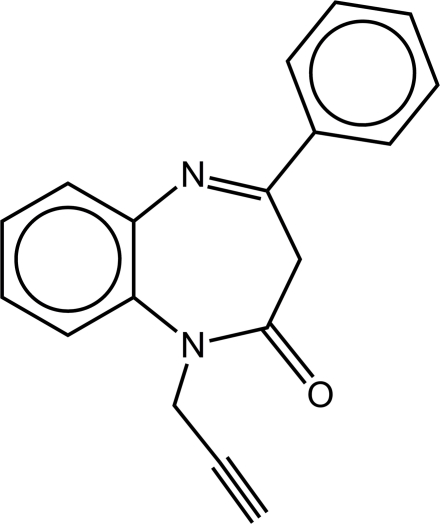


## Experimental

### 

#### Crystal data

C_18_H_14_N_2_O
*M* *_r_* = 274.31
Monoclinic, 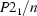

*a* = 8.2574 (14) Å
*b* = 18.961 (3) Å
*c* = 9.0914 (15) Å
β = 102.962 (4)°
*V* = 1387.1 (4) Å^3^
*Z* = 4
Mo *K*α radiation
μ = 0.08 mm^−1^
*T* = 150 K
0.12 × 0.08 × 0.04 mm

#### Data collection

Bruker X8 Kappa CCD APEX II diffractometer
Absorption correction: multi-scan (*SADABS*; Sheldrick, 1997 ▶) *T*_min_ = 0.990, *T*_max_ = 0.997
11049 measured reflections
5228 independent reflections
3621 reflections with *I* > 2σ(*I*)
*R*_int_ = 0.034

#### Refinement

*R*[*F* ^2^ > 2σ(*F* ^2^)] = 0.052
*wR*(*F* ^2^) = 0.143
*S* = 1.05
5228 reflections
190 parameters
H-atom parameters constrained
Δρ_max_ = 0.45 e Å^−3^
Δρ_min_ = −0.25 e Å^−3^

### 

Data collection: *APEX2* (Bruker, 2006 ▶); cell refinement: *SAINT-Plus* (Bruker, 2005 ▶); data reduction: *SAINT-Plus*; program(s) used to solve structure: *SHELXTL* (Sheldrick, 2008 ▶); program(s) used to refine structure: *SHELXTL*; molecular graphics: *DIAMOND* (Brandenburg, 2009 ▶); software used to prepare material for publication: *SHELXTL*.

## Supplementary Material

Crystal structure: contains datablock(s) global, I. DOI: 10.1107/S1600536811027371/tk2762sup1.cif

Structure factors: contains datablock(s) I. DOI: 10.1107/S1600536811027371/tk2762Isup2.hkl

Supplementary material file. DOI: 10.1107/S1600536811027371/tk2762Isup3.cml

Additional supplementary materials:  crystallographic information; 3D view; checkCIF report

## Figures and Tables

**Table 1:** Hydrogen-bond geometry (Å, °)

*D*—H⋯*A*	*D*—H	H⋯*A*	*D*⋯*A*	*D*—H⋯*A*
C1—H1*A*⋯O1^i^	0.99	2.14	3.1074 (15)	166
C3—H3⋯N2^ii^	0.95	2.58	3.4269 (18)	149

Symmetry codes: (i) 
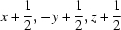
; (ii) 
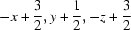
.

**Fig. 1.**
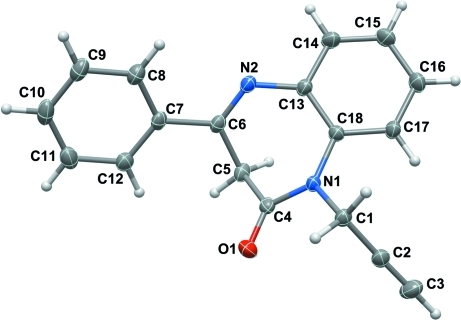
Asymmetric unit of the title compound showing all non-hydrogen atoms represented as thermal ellipsoids drawn at the 50% probability level and hydrogen atoms as small spheres with arbitrary radii.

**Fig. 2.**
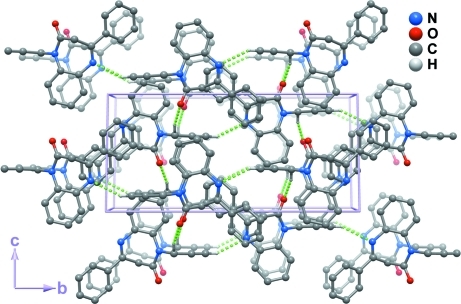
Crystal packing of the title compound viewed in perspective along the [100] direction of the unit cell. C—H···(N,O) weak hydrogen bonds are represented as dashed green lines. H-atoms not involved in hydrogen bonding interactions have been omitted for clarity.

## supplementary crystallographic information

### Comment

Benzodiazepine derivatives are an important class of heterocyclic compounds in the field of drugs, pharmaceuticals and synthetic organic chemistry (Bird, 1996; Wolff, 1996), as they show antiviral (Di Braccio *et al.*, 2001), analgesic (Di Braccio *et al.*, 1990), and antipsychotic (Kavita *et al.*, 1988) activities. These compounds are used worldwide as anticonvulsant agents (Sieghart & Schuster, 1984) or as sedative or hypnotics (Goetzke *et al.*, 1983; Ahmed *et al.*, 1983). Examples of well known diazepines are Alprazolam, Diazepam and Flunitrazepam (Wolff, 1996). Their pharmacological effects come from the activation of the benzodiazepine receptor which interacts with the GABA recognition site (Meldrum & Chapman, 1986). Research in this area is highly active being directed towards the synthesis of compounds with enhanced pharmacological activity. Following the research efforts from some of us concerning novel synthetic pathways of new benzodiazepines (Aatif *et al.*, 2000; Baouid *et al.*, 2001; Boudina *et al.*, 2007), and our interest on the structural features of organic crystals (Fernandes *et al.*, 2011; Amarante, Figueiredo *et al.*, 2009; Amarante, Gonçalves & Almeida Paz , 2009; Paz & Klinowski, 2003; Paz *et al.*, 2002), here we wish to report the synthesis *via* phase transfer catalysis and the crystallographic studies of the title compound (I).

The asymmetric unit is composed of a whole molecular moiety of I (Fig. 1). All atoms are distributed over four medium planes (see Table 1 for details), which converge in the diazepine ring. The plane of the substituent aromatic ring is extended to the imine group from the diazepine moiety (plane A) and subtends an angle of 71.78 (4)° with the amide plane (C). The plane of the benzo ring (B) subtends, on the other hand, two almost similar angles with the previously described planes [41.76 (4)° with plane A and 40.75 (4)° with plane C]. The plane of the propargyl substituent (D) is almost perpendicular with that of the amide group [87.81 (8)°].

The crystal packing (Fig. 2) features weak supramolecular interactions (see Table 2 for details), namely the C—H and CH_2_ groups of the propargyl moiety interact with N2 from the imine and O1 from the amide of neighbouring molecules, respectively.

### Experimental

Melting points were taken in an open capillary tube on a Buchi 510 apparatus and are uncorrected. The FT—IR spectrum was obtained from KBr pellets using a Bruker Tensor 27 spectrophotometer. NMR Spectra were recorded with the following instruments: ^1^H, Bruker AC-300; ^13^C, Bruker AC-75. TMS was used as an internal reference. Mass spectra were recorded using a Jeol JMS DX 300 instrument. Column chromatography was carried out using E-Merck silica gel 60F_254_. All reagents were purchased from commercial sources and were used without further purification.

The precursor, 4-phenyl-2,3-dihydro-1*H*-1,5-benzodiazepin-2-one (II), was prepared following literature procedures (Nardi *et al.*, 1973) by refluxing *o*-phenylenediamine and ethyl benzoylacetate for 2 h in xylene.

A mixture of 1 g (4.6 mmol) of II, 0.43 g (2.3 mmol) of benzyltriethylammonium chloride (TBA-Cl) and 3 ml of a 50% sodium hydroxide aqueous solution in benzene (25 ml) was stirred at ambient temperature. After 15 min, propargyl bromide was added slowly. After 6 h of stirring at 298 K, the reaction mixture was diluted with water (30 ml). The organic layer was extracted with benzene (3 × 10 ml), dried over anhydrous sodium sulfate and evaporated under vacuum. The title compound was isolated by column chromatography on silica gel using hexane/ethyl acetate as eluent. The solid product was recrystallized in dichloromethane to give yellow crystals of I. Yield: 96%. Melting point: 438–440 K.

FT–IR (KBr): 3259(*m*), 3060(*w*), 2984(*m*), 1659(*vs*), 1602(*s*), 1586(*w*), 1570(*m*), 1496(*w*), 1479(*s*), 1452(*s*), 1431(*m*), 1379(*s*), 1362(*w*), 1321(*w*), 1307(*m*), 1293(*w*), 1279(*m*), 1262(*m*), 1211(*m*), 1162(*w*), 1014(*m*), 958(*m*), 774(*s*), 688(*m*), 662(*w*), 639(*w*), 598(*m*), 484(*w*), 426(*w*) cm^-1^. ^1^H NMR (300 MHz, CDCl_3_): 7.25-8.14 (9H, Ar-H), 4.19 and 4.27 (AB system, d, *J*= 17.7 Hz, 2H, N-CH_2_-C), 3.04 and 4.76 (AB system, d, *J*=12 Hz, 2H, CH_2_-CO-N), 2.32 (t, *J*= 2.25 Hz, 1H, HC≡C) ppm.^13^C NMR (75 MHz, CDCl_3_): 165 (1C, CO), 160.0 (1C, Ph-C=N), 140.9, 136.9, 133.3, 130.5, 128.1, 127.1, 126.7, 125.7, 125.1, 120.9 (12C, Ar-C), 78.5 (1C, HC≡ C), 71.9 (1C, HC≡C), 39.1 (1C, CH_2_-CO-N), 36.9 (1C, N-CH_2_-C) ppm. MS (EI, *m/z*): 275 [*M*+H]^+^.

### Refinement

Hydrogen atoms bound to carbon were placed at their idealized positions and were included in the final structural model in riding-motion approximation with C—H = 0.95 Å (aromatic and acetylenic), and C—H = 0.99 Å (aliphatic —CH_2_—). The isotropic thermal displacement parameters for these atoms were fixed at 1.2×*U*_eq_ of the respective parent carbon atom.

### Crystal data

**Table:**

C_18_H_14_N_2_O	*F*(000) = 576
*M**_r_* = 274.31	*D*_x_ = 1.314 Mg m^−^^3^
Monoclinic, *P*2_1_/*n*	Mo *K*α radiation, λ = 0.71073 Å
Hall symbol: -P 2yn	Cell parameters from 2335 reflections
*a* = 8.2574 (14) Å	θ = 2.5–32.7°
*b* = 18.961 (3) Å	µ = 0.08 mm^−^^1^
*c* = 9.0914 (15) Å	*T* = 150 K
β = 102.962 (4)°	Block, yellow
*V* = 1387.1 (4) Å^3^	0.12 × 0.08 × 0.04 mm
*Z* = 4	

### Data collection

**Table:**

Bruker X8 Kappa CCD APEX II diffractometer	5228 independent reflections
Radiation source: fine-focus sealed tube	3621 reflections with *I* > 2σ(*I*)
graphite	*R*_int_ = 0.034
ω / φ scans	θ_max_ = 33.1°, θ_min_ = 3.7°
Absorption correction: multi-scan (*SADABS*; Sheldrick, 1997)	*h* = −12→11
*T*_min_ = 0.990, *T*_max_ = 0.997	*k* = −24→29
11049 measured reflections	*l* = −10→13

### Refinement

**Table:**

Refinement on *F*^2^	Primary atom site location: structure-invariant direct methods
Least-squares matrix: full	Secondary atom site location: difference Fourier map
*R*[*F*^2^ > 2σ(*F*^2^)] = 0.052	Hydrogen site location: inferred from neighbouring sites
*wR*(*F*^2^) = 0.143	H-atom parameters constrained
*S* = 1.05	*w* = 1/[σ^2^(*F*_o_^2^) + (0.0689*P*)^2^ + 0.095*P*] where *P* = (*F*_o_^2^ + 2*F*_c_^2^)/3
5228 reflections	(Δ/σ)_max_ < 0.001
190 parameters	Δρ_max_ = 0.45 e Å^−^^3^
0 restraints	Δρ_min_ = −0.25 e Å^−^^3^

### Special details

**Table:**

Geometry. All e.s.d.'s (except the e.s.d. in the dihedral angle between two l.s. planes) are estimated using the full covariance matrix. The cell e.s.d.'s are taken into account individually in the estimation of e.s.d.'s in distances, angles and torsion angles; correlations between e.s.d.'s in cell parameters are only used when they are defined by crystal symmetry. An approximate (isotropic) treatment of cell e.s.d.'s is used for estimating e.s.d.'s involving l.s. planes.
Refinement. Refinement of *F*^2^ against ALL reflections. The weighted *R*-factor *wR* and goodness of fit *S* are based on *F*^2^, conventional *R*-factors *R* are based on *F*, with *F* set to zero for negative *F*^2^. The threshold expression of *F*^2^ > σ(*F*^2^) is used only for calculating *R*-factors(gt) *etc*. and is not relevant to the choice of reflections for refinement. *R*-factors based on *F*^2^ are statistically about twice as large as those based on *F*, and *R*- factors based on ALL data will be even larger.

### Fractional atomic coordinates and isotropic or equivalent isotropic displacement parameters (Å^2^)

**Table:**

	*x*	*y*	*z*	*U*_iso_*/*U*_eq_	
N1	0.81747 (12)	0.20787 (5)	0.66080 (10)	0.01806 (19)	
N2	0.80660 (13)	0.05512 (5)	0.73647 (10)	0.0212 (2)	
O1	0.73372 (11)	0.20477 (5)	0.40627 (9)	0.0263 (2)	
C1	0.92473 (14)	0.26835 (6)	0.64614 (12)	0.0203 (2)	
H1A	1.0213	0.2687	0.7333	0.024*	
H1B	0.9675	0.2624	0.5535	0.024*	
C2	0.83874 (16)	0.33625 (6)	0.63915 (12)	0.0247 (2)	
C3	0.7724 (2)	0.39109 (7)	0.63764 (16)	0.0338 (3)	
H3	0.7187	0.4355	0.6364	0.041*	
C4	0.72159 (14)	0.18209 (6)	0.52947 (11)	0.0191 (2)	
C5	0.60888 (14)	0.12224 (6)	0.54917 (13)	0.0218 (2)	
H5A	0.5294	0.1118	0.4527	0.026*	
H5B	0.5453	0.1343	0.6260	0.026*	
C6	0.72038 (14)	0.05952 (6)	0.59981 (12)	0.0197 (2)	
C7	0.73684 (14)	0.00310 (6)	0.49079 (12)	0.0203 (2)	
C8	0.84549 (16)	−0.05308 (6)	0.53927 (14)	0.0250 (2)	
H8	0.9068	−0.0547	0.6410	0.030*	
C9	0.86457 (17)	−0.10638 (7)	0.44028 (15)	0.0289 (3)	
H9	0.9375	−0.1446	0.4751	0.035*	
C10	0.77807 (19)	−0.10440 (7)	0.29068 (15)	0.0318 (3)	
H10	0.7916	−0.1410	0.2231	0.038*	
C11	0.67212 (19)	−0.04871 (7)	0.24087 (14)	0.0318 (3)	
H11	0.6135	−0.0468	0.1383	0.038*	
C12	0.65061 (17)	0.00474 (7)	0.34008 (13)	0.0263 (3)	
H12	0.5767	0.0426	0.3048	0.032*	
C13	0.80668 (15)	0.11000 (6)	0.83976 (12)	0.0206 (2)	
C14	0.81461 (17)	0.09009 (6)	0.99013 (13)	0.0265 (3)	
H14	0.8139	0.0414	1.0143	0.032*	
C15	0.82341 (17)	0.13908 (7)	1.10369 (13)	0.0285 (3)	
H15	0.8261	0.1241	1.2039	0.034*	
C16	0.82834 (16)	0.21074 (7)	1.07035 (13)	0.0259 (2)	
H16	0.8353	0.2449	1.1480	0.031*	
C17	0.82299 (15)	0.23177 (6)	0.92394 (12)	0.0221 (2)	
H17	0.8267	0.2806	0.9020	0.026*	
C18	0.81219 (14)	0.18262 (6)	0.80699 (11)	0.0187 (2)	

### Atomic displacement parameters (Å^2^)

**Table:**

	*U*^11^	*U*^22^	*U*^33^	*U*^12^	*U*^13^	*U*^23^
N1	0.0243 (5)	0.0140 (4)	0.0163 (4)	−0.0031 (4)	0.0055 (3)	−0.0011 (3)
N2	0.0281 (5)	0.0145 (4)	0.0223 (4)	0.0000 (4)	0.0083 (4)	−0.0004 (3)
O1	0.0319 (5)	0.0288 (5)	0.0172 (3)	−0.0007 (4)	0.0036 (3)	0.0012 (3)
C1	0.0248 (5)	0.0163 (5)	0.0207 (4)	−0.0036 (4)	0.0071 (4)	−0.0004 (4)
C2	0.0341 (6)	0.0196 (5)	0.0213 (5)	−0.0056 (5)	0.0083 (4)	0.0002 (4)
C3	0.0461 (8)	0.0222 (6)	0.0355 (6)	0.0045 (6)	0.0145 (6)	0.0060 (5)
C4	0.0209 (5)	0.0165 (5)	0.0192 (4)	0.0034 (4)	0.0033 (4)	−0.0012 (4)
C5	0.0213 (5)	0.0176 (5)	0.0258 (5)	−0.0010 (4)	0.0038 (4)	−0.0033 (4)
C6	0.0227 (5)	0.0138 (5)	0.0241 (5)	−0.0023 (4)	0.0082 (4)	−0.0015 (4)
C7	0.0241 (5)	0.0146 (5)	0.0242 (5)	−0.0042 (4)	0.0092 (4)	−0.0029 (4)
C8	0.0265 (6)	0.0189 (5)	0.0310 (5)	0.0004 (4)	0.0094 (4)	−0.0032 (5)
C9	0.0323 (6)	0.0204 (6)	0.0379 (6)	0.0021 (5)	0.0162 (5)	−0.0042 (5)
C10	0.0422 (8)	0.0240 (6)	0.0355 (6)	−0.0056 (6)	0.0224 (6)	−0.0095 (5)
C11	0.0438 (8)	0.0296 (7)	0.0243 (5)	−0.0063 (6)	0.0126 (5)	−0.0053 (5)
C12	0.0350 (7)	0.0198 (5)	0.0252 (5)	−0.0019 (5)	0.0088 (5)	−0.0005 (4)
C13	0.0263 (5)	0.0158 (5)	0.0208 (4)	0.0005 (4)	0.0074 (4)	−0.0009 (4)
C14	0.0387 (7)	0.0197 (5)	0.0229 (5)	0.0008 (5)	0.0105 (5)	0.0036 (4)
C15	0.0397 (7)	0.0290 (6)	0.0190 (5)	0.0015 (5)	0.0110 (4)	0.0023 (5)
C16	0.0346 (6)	0.0254 (6)	0.0194 (4)	0.0000 (5)	0.0099 (4)	−0.0038 (4)
C17	0.0299 (6)	0.0170 (5)	0.0204 (4)	−0.0007 (4)	0.0078 (4)	−0.0025 (4)
C18	0.0224 (5)	0.0174 (5)	0.0169 (4)	−0.0002 (4)	0.0060 (4)	0.0001 (4)

### Geometric parameters (Å, °)

**Table:**

N1—C4	1.3665 (13)	C8—H8	0.9500
N1—C18	1.4226 (13)	C9—C10	1.388 (2)
N1—C1	1.4731 (14)	C9—H9	0.9500
N2—C6	1.2886 (14)	C10—C11	1.381 (2)
N2—C13	1.4017 (14)	C10—H10	0.9500
O1—C4	1.2246 (13)	C11—C12	1.3942 (17)
C1—C2	1.4648 (17)	C11—H11	0.9500
C1—H1A	0.9900	C12—H12	0.9500
C1—H1B	0.9900	C13—C14	1.4056 (16)
C2—C3	1.1739 (19)	C13—C18	1.4115 (16)
C3—H3	0.9500	C14—C15	1.3785 (17)
C4—C5	1.5037 (16)	C14—H14	0.9500
C5—C6	1.5112 (16)	C15—C16	1.3947 (18)
C5—H5A	0.9900	C15—H15	0.9500
C5—H5B	0.9900	C16—C17	1.3807 (16)
C6—C7	1.4851 (15)	C16—H16	0.9500
C7—C12	1.3956 (16)	C17—C18	1.4015 (15)
C7—C8	1.3988 (17)	C17—H17	0.9500
C8—C9	1.3855 (17)		
			
C4—N1—C18	124.24 (10)	C8—C9—C10	120.56 (12)
C4—N1—C1	116.21 (9)	C8—C9—H9	119.7
C18—N1—C1	119.45 (9)	C10—C9—H9	119.7
C6—N2—C13	120.99 (10)	C11—C10—C9	119.40 (12)
C2—C1—N1	113.12 (10)	C11—C10—H10	120.3
C2—C1—H1A	109.0	C9—C10—H10	120.3
N1—C1—H1A	109.0	C10—C11—C12	120.45 (12)
C2—C1—H1B	109.0	C10—C11—H11	119.8
N1—C1—H1B	109.0	C12—C11—H11	119.8
H1A—C1—H1B	107.8	C11—C12—C7	120.53 (12)
C3—C2—C1	178.08 (13)	C11—C12—H12	119.7
C2—C3—H3	180.0	C7—C12—H12	119.7
O1—C4—N1	121.55 (11)	N2—C13—C14	116.43 (10)
O1—C4—C5	123.62 (10)	N2—C13—C18	125.31 (10)
N1—C4—C5	114.75 (9)	C14—C13—C18	118.07 (10)
C4—C5—C6	106.21 (9)	C15—C14—C13	122.04 (11)
C4—C5—H5A	110.5	C15—C14—H14	119.0
C6—C5—H5A	110.5	C13—C14—H14	119.0
C4—C5—H5B	110.5	C14—C15—C16	119.54 (11)
C6—C5—H5B	110.5	C14—C15—H15	120.2
H5A—C5—H5B	108.7	C16—C15—H15	120.2
N2—C6—C7	118.90 (10)	C17—C16—C15	119.64 (11)
N2—C6—C5	120.78 (10)	C17—C16—H16	120.2
C7—C6—C5	120.28 (9)	C15—C16—H16	120.2
C12—C7—C8	118.44 (11)	C16—C17—C18	121.48 (11)
C12—C7—C6	122.43 (11)	C16—C17—H17	119.3
C8—C7—C6	119.11 (10)	C18—C17—H17	119.3
C9—C8—C7	120.60 (12)	C17—C18—C13	119.22 (10)
C9—C8—H8	119.7	C17—C18—N1	118.34 (10)
C7—C8—H8	119.7	C13—C18—N1	122.33 (10)
			
C4—N1—C1—C2	84.64 (12)	C10—C11—C12—C7	0.6 (2)
C18—N1—C1—C2	−91.95 (12)	C8—C7—C12—C11	0.32 (18)
C18—N1—C4—O1	−178.39 (11)	C6—C7—C12—C11	178.80 (11)
C1—N1—C4—O1	5.21 (16)	C6—N2—C13—C14	144.00 (12)
C18—N1—C4—C5	−1.33 (15)	C6—N2—C13—C18	−41.08 (17)
C1—N1—C4—C5	−177.73 (9)	N2—C13—C14—C15	176.89 (12)
O1—C4—C5—C6	106.80 (12)	C18—C13—C14—C15	1.6 (2)
N1—C4—C5—C6	−70.19 (12)	C13—C14—C15—C16	−1.4 (2)
C13—N2—C6—C7	174.26 (10)	C14—C15—C16—C17	0.5 (2)
C13—N2—C6—C5	−3.54 (17)	C15—C16—C17—C18	0.18 (19)
C4—C5—C6—N2	75.83 (13)	C16—C17—C18—C13	−0.01 (18)
C4—C5—C6—C7	−101.94 (11)	C16—C17—C18—N1	−176.21 (11)
N2—C6—C7—C12	−178.58 (11)	N2—C13—C18—C17	−175.69 (11)
C5—C6—C7—C12	−0.77 (17)	C14—C13—C18—C17	−0.85 (17)
N2—C6—C7—C8	−0.12 (16)	N2—C13—C18—N1	0.36 (18)
C5—C6—C7—C8	177.70 (11)	C14—C13—C18—N1	175.20 (11)
C12—C7—C8—C9	−1.09 (18)	C4—N1—C18—C17	−140.25 (11)
C6—C7—C8—C9	−179.61 (11)	C1—N1—C18—C17	36.05 (15)
C7—C8—C9—C10	0.98 (19)	C4—N1—C18—C13	43.68 (17)
C8—C9—C10—C11	−0.1 (2)	C1—N1—C18—C13	−140.03 (11)
C9—C10—C11—C12	−0.7 (2)		

### Hydrogen-bond geometry (Å, °)

**Table:**

*D*—H···*A*	*D*—H	H···*A*	*D*···*A*	*D*—H···*A*
C1—H1A···O1^i^	0.99	2.14	3.1074 (15)	166
C3—H3···N2^ii^	0.95	2.58	3.4269 (18)	149

Symmetry codes: (i) *x*+1/2, −*y*+1/2, *z*+1/2; (ii) −*x*+3/2, *y*+1/2, −*z*+3/2.

### Table 2 Medium planes existent in the molecular unit of the title compound

**Table:**

Plane	Atoms	Largest deviation/Å
A	C5 to C12 plus N2	-0.019 (1)
B	C13 to C18 plus N1, N2	-0.039 (1)
C	C1 to C5 plus O1, N1, C18	-0.026 (1)
D	C1 to C3 plus N1	-0.014 (1)
